# UNMET NEEDS AND HEALTHCARE SERVICE UTILIZATION AMONG OLDER ADULTS: A SYSTEMATIC REVIEW

**DOI:** 10.1093/geroni/igae098.3919

**Published:** 2024-12-31

**Authors:** Maham Khan, Laura Sands, Cozette Comer, Jackson Hoch

**Affiliations:** Virginia Tech., Blacksburg, Virginia, United States; Virginia Tech, Blacksburg, Virginia, United States; Virginia Tech, Virginia Tech, Virginia, United States; Virginia Tech, Virginia Tech, Virginia, United States

## Abstract

Unmet needs refer to the absence of necessary assistance for completing Activities of Daily Living (ADLs), and they are associated with severe health consequences, such as hospitalizations, emergency room visits, nursing home placement, and mortality among older adults. The high cost of unmet needs pose challenges at both individual and systemic levels. While the prior literature has focused on immediate consequences of unmet needs (such as going without bathing or toileting), this systematic review consolidates evidence to assess the impact of unmet needs on healthcare utilization and emphasizes the importance of improving community-based care to mitigate (costly) consequences. A comprehensive search of multiple databases, including Scopus, Web of Science, PubMed, and CINAHL, using appropriate keywords (unmet needs, acute healthcare utilization) identified 6,475 studies. After a rigorous screening process, eight studies met the inclusion criteria for analysis, focusing on non-institutionalized older adults in the U.S. with at least one ADL difficulty. Results show that individuals with unmet needs experience double the hospitalization rates compared to those with met needs. Emergency room visits are also 31% higher, especially among those with unmet self-care needs. From the hospital deaths, 74 % report to have experienced unmet needs at end-of-life. Sociodemographic factors, such as older age, being minority race, living alone, and cognitive impairment further exacerbate the risks acute healthcare utilization. Addressing unmet needs is crucial not only for improving quality of life among older adults but also for alleviating the strain on healthcare resources through targeted policy interventions.

